# Impact of demographic, clinical, and viral factors on severe influenza in young children in Fuzhou

**DOI:** 10.3389/fpubh.2026.1718973

**Published:** 2026-06-02

**Authors:** Xiaoyan Zheng, Ling Yao, Zhijie Wu, Xiaoyang Zhang, Youqiong Xu

**Affiliations:** 1 The Affiliated Fuzhou Center for Disease Control and Prevention of Fujian Medical University, Fuzhou, China; 2 The School of Public Health, Fujian Medical University, Fuzhou, China; 3 Fujian Medical Uvniversity, Fuzhou, China

**Keywords:** case–control study, child, immune vaccination, influenza, risk factor, severe case

## Abstract

**Background:**

Identify risk factors for severe influenza in children under 5 years of age in the Fuzhou region to provide evidence-based support for early identification of high-risk patients, optimization of Pediatric Intensive Care Unit (PICU) resource allocation, and development of targeted intervention strategies.

**Methods:**

A total of 386 severe influenza cases and 1,544 non-severe cases among children under 5 years old in Fuzhou City were collected from January 2015 to January 2025. The non-severe cases were matched for the same period, same region, parental education level, and age (±1 year). A retrospective case–control study design was employed to analyze demographic characteristics, clinical symptoms, laboratory indicators, medical history, and viral subtypes between the two groups. Multivariate logistic regression analysis was used to identify independent risk factors for severe influenza.

**Results:**

Multivariate analysis revealed that male gender, children living in dispersed communities, obesity, rural residence, lack of influenza vaccination, time from onset to presentation >2 days, time from onset to antiviral treatment >2 days, persistent high fever >3 days, tachypnea/dyspnea, altered mental status/ convulsions, elevated neutrophil-to-lymphocyte ratio (NLR), high C-reactive protein (CRP) levels, low albumin levels, history of chronic respiratory disease, history of metabolic and endocrine disorders, and infection with influenza A (H1N1) virus were all risk factors for severe influenza in children under 5 years of age (all *p* < 0.05). The median recovery time in the severe group was 19 days, significantly longer than the 10 days in the non-severe group (*p* < 0.001).

**Conclusion:**

The occurrence of severe influenza in children under 5 years of age is influenced by multiple factors, including demographic characteristics, clinical features, laboratory indicators, past medical history, and viral subtype. Efforts should focus on strengthening prevention and control in key populations, increasing vaccination rates, optimizing the allocation of medical resources, and enhancing primary care capabilities to reduce the incidence of severe influenza in children and improve outcomes.

## 1 Introduction

Influenza is an acute respiratory infectious disease characterized by high incidence and rapid transmission, making it prone to pandemics and outbreaks (1). Clinical symptoms include fever, muscle aches, and headaches, potentially accompanied by complications affecting multiple systems such as respiratory, digestive, and neurological (2). Severe cases require hospitalization, sometimes necessitating admission to pediatric intensive care units (ICUs). Studies indicate that approximately 25% of children contract influenza during each seasonal epidemic (3). Children under 5 years old constitute a high-incidence group for influenza and are also at high risk for severe illness. It is estimated that tens of thousands of children under five worldwide die annually from influenza-related complications (4, 5). Despite advances in diagnosing and treating influenza virus infections in children, the high mortality rate associated with severe influenza pneumonia—particularly acute necrotizing pneumonia (ANE)—has not significantly decreased (6).

Identifying factors that may lead to severe influenza hospitalization, ICU admission, and death is crucial for implementing optimal prevention and control measures in children. Previous influenza-related studies often employed inconsistent criteria for defining influenza and its severity, and in many cases, these criteria proved difficult to apply to pediatric populations. Research indicates multiple factors influence pediatric influenza outcomes, including preterm birth, age ≤2 years, immunological, genetic, and viral factors. However, key determinants of influenza progression to severe disease remain poorly quantified, leading to controversy regarding methods for assessing disease incidence and severity in pediatric populations (7).

This study collected survey data from 386 severe influenza cases and 1,544 non-severe cases among children under 5 years old in Fuzhou City between January 2015 and January 2025. By analyzing risk factors for severe illness, it provides evidence-based support for early identification of high-risk children, optimization of Pediatric Intensive Care Unit (PICU) resource allocation, and development of targeted intervention strategies. Additionally, it offers data support for health administrative departments in formulating priority vaccination lists for seasonal influenza vaccines.

## 2 Methods

### 2.1 Study subjects

Patient data were obtained from the China Influenza Surveillance Information System and the China Disease Prevention and Control Information System. The study included 386 severe influenza cases in children under 5 years of age who were diagnosed in Fuzhou from January 2015 to January 2025. For each severe case, four nonsevere cases were matched by the same period, region, parental education, and age (±1 year), forming a nonsevere group of 1,544 cases. This research has been reviewed and approved by the ethical research board committee of Fuzhou Center for Disease Control and Prevention (Approval No. IRB [2023] No. (02)). The need for individual informed consents is waived by the institution/ review board due to the face that exclusively utilized anonymized aggregated data was used and did not involve any individual subjects.

The inclusion criteria for patients were as follows: met the confirmed and severe influenza diagnostic criteria and were verified by clinicians. The exclusion criteria for children were uncooperative families, incomplete data, congenital immunodeficiencies, or other serious illnesses.

### 2.2 Case definitions

In accordance with the “Influenza Diagnosis and Treatment Plan (2020 Edition)” (http://www.nhc.gov.cn/ylyjs/s7653p/202011/a943c67d55c74e589d23c81d65b5e221.shtml), a confirmed case was defined as a patient who presented with clinical symptoms of influenza and tested positive for influenza virus nucleic acid. A severe case was defined as a confirmed case with any of the following: (1) fever >3 days with severe cough or chest pain; (2) rapid breathing or dyspnea; (3) altered mental status or convulsions; (4) severe vomiting or diarrhea; or (5) pneumonia.

### 2.3 Survey method

The survey covered five aspects: (1) demographic characteristics: sex, age, and place of residence; (2) clinical symptoms: fever, cough, tachypnea/dyspnea; (3) laboratory tests: WBC, lymphocyte, and neutrophil counts; (4) medical history: history of chronic respiratory diseases, metabolic and endocrine diseases, etc.; and (5) pathogen types.

### 2.4 Variable definitions

#### 2.4.1 Obesity

Body mass index (BMI) was calculated as weight (kg)/height (m^2^). Obesity was defined as BMI-for-age ≥ the 95th percentile according to the national growth reference for Chinese children (ref), stratified by sex.

#### 2.4.2 Rural/urban residence

Rural residence was defined as living in townships or administrative villages, whereas urban residence referred to municipal districts and subdistricts, based on registered residential addresses in the surveillance system.

#### 2.4.3 Laboratory indices

NLR was calculated as absolute neutrophil count divided by lymphocyte count. ‘High NLR’ was defined as NLR ≥ 1.52 based on the optimal Youden index from ROC analysis. ‘High CRP’ was defined as CRP ≥ 10 mg/L, and ‘low albumin’ as serum albumin <40 g/L.”

### 2.5 Definition of care setting

Children were categorized by their primary daytime care arrangement at the time of illness. “Children not enrolled in child-care institutions” (also referred to as “home-care children”) were defined as those who did not attend any formal daycare center, kindergarten, or preschool, and were primarily cared for at home by family members. This group was compared with “children enrolled in child-care institutions” who attended such facilities regularly.

### 2.6 Etiological testing

Oropharyngeal swab samples were collected, placed into 3 mL noninactivated virus sampling tubes, mixed and left to stand. Using the bioperfectus-brand influenza virus nucleic acid detection kit, RT–PCR with fluorescent probes was applied to detect influenza A and B viruses in the samples. The procedure included nucleic acid extraction, reaction system preparation (on ice to prevent enzyme inactivation), and precise measurement of reagent volumes and concentrations. For example, the reaction mixture consisted of 1 μL of DNA template, 1 μL each of forward and reverse primers (10 μM), 2 μL of dNTPs (2.5 mM), 2.5 μL of 10 × PCR buffer, 0.2 μL of Taq enzyme (5 U/μl), and ddH2O to make up to 25 μL. Finally, the PCR program was set for amplification. The sensitivity of the kit for detecting H1N1, H3N2, and influenza B viruses was 95, 93, and 93%, respectively, and the specificity was 93, 94, and 94%, respectively.

### 2.7 Statistical analysis

Data analysis was performed via SPSS 26.0. Count data were analyzed with the *χ*^2^ test or Fisher’s exact test, and measurement data were analyzed with the *t* test or *Z* test. Variables significant in the univariate analysis were included in the multivariate logistic regression. Statistical significance was set at *p* < 0.05.

## 3 Results

### 3.1 Demographic characteristics of the two groups

From January 2015 to January 2025, a total of 386 severe influenza cases among children under 5 years old were reported in Fuzhou city. In terms of sex, the proportion of males was greater in the severe group (56.99%) than in the nonsevere group (45.40%). In terms of population characteristics, the proportion of noninstitutionalized children was greater in the severe group (59.59%) than in the nonsevere group (48.06%). In terms of obesity, the proportion was greater in the severe group (21.24%) than in the nonsevere group (11.27%). With respect to place of residence, the proportion of children from rural areas was greater in the severe group (38.34%) than in the nonsevere group (24.48%). In terms of vaccination, the proportion of vaccinated children was lower in the severe group (22.80%) than in the nonsevere group (37.44%). The differences in the above aspects were statistically significant (*p* < 0.001). However, there was no statistically significant difference between the two groups in terms of age (*p* > 0.05), as shown in Table 1.

**Table 1:** Comparison of demographic characteristics between severe and nonsevere cases of influenza in Fuzhou [*n* (%)].

Characteristic	Contents	Severe group (*n* = 386)	Non severe group (*n* = 1,544)	*χ* ^2^	*p*
Gender	Male	220 (56.99%)	701 (45.40%)	16.636	<0.001
	Female	166 (43.01%)	843 (54.60%)		
Age	≤3	213 (55.18%)	813 (52.66%)	0.791	0.374
	>3	173 (44.82%)	731 (47.34%)		
Population characteristics	Children not enrolled in child-care institutions (home-care)	230 (59.59%)	742 (48.06%)	16.417	<0.001
	Nursery children	156 (40.41%)	802 (51.94%)		
Obesity or not	Yes	82 (21.24%)	174 (11.27%)	26.702	<0.001
	No	304 (78.76%)	1,370 (88.73%)		
Current address attribution	Rural area	148 (38.34%)	378 (24.48%)	29.921	<0.001
	Urban area	238 (61.66%)	1,166 (75.52%)		
Vaccinate	Yes	88 (22.80%)	578 (37.44%)	29.275	<0.001
	No	298 (77.20%)	966 (62.56%)		

### 3.2 Clinical symptoms of the two groups

The proportions of patients in the severe group with an interval of more than 2 days between the onset of illness and the first medical visit, more than 2 days between the onset of illness and the initiation of antiviral treatment, a fever lasting more than 3 days, tachypnea/breathing difficulty, and altered mental status/seizures were 29.02, 26.17, 26.42, 27.20, and 24.35%, respectively. These proportions were significantly greater than those in the nonsevere group, which were 17.49, 16.13, 16.32, 14.64, and 13.47%, respectively. The differences were statistically significant (*p* < 0.001). However, there were no statistically significant differences in other clinical symptoms between the two groups (*p* > 0.05), as shown in Table 2.

**Table 2:** Comparison of clinical symptoms between severe and nonsevere cases of influenza in Fuzhou [*n* (%)].

Typical symptoms	Severe group (*n* = 386)	Non severe group (*n* = 1,544)	*χ* ^2^	*p*
Time from onset to treatment > 2d	112 (29.02%)	270 (17.49%)	25.853	<0.001
Time from onset to antiviral treatment > 2d	101 (26.17%)	249 (16.13%)	20.962	<0.001
High fever duration > 3d	102 (26.42%)	252 (16.32%)	21.047	<0.001
Cough/expectoration	252 (65.28%)	1,003 (64.96%)	0.014	0.905
Sore throat	172 (44.56%)	626 (40.54%)	2.053	0.152
Dizziness/headache	61 (15.80%)	201 (13.02%)	2.041	0.153
Vomit	52 (13.47%)	188 (12.18%)	0.476	0.490
Asthma	29 (7.51%)	82 (5.31%)	2.762	0.096
Shortness of breath/dyspnea	105 (27.20%)	226 (14.64%)	34.310	<0.001
Nasal congestion	125 (32.38%)	452 (29.27%)	1.424	0.233
Runny nose	113 (29.27%)	397 (25.71%)	2.015	0.156
Change of mind/convulsion	94 (24.35%)	208 (13.47%)	27.698	<0.001
General fatigue	76 (19.69%)	284 (18.39%)	0.341	0.559
Abdominal pain/diarrhea	38 (9.84%)	116 (7.51%)	2.286	0.131
Muscle soreness	95 (24.61%)	327 (21.18%)	2.130	0.144
Chills	57 (14.77%)	183 (11.85%)	2.409	0.121

Mediation analysis revealed that “time from onset to antiviral treatment” partially mediated the relationship between “time from onset to medical consultation” and “severe influenza.” The mediating effect was significant but of small magnitude (Table 3).

**Table 3:** Regression analysis results of the mediating effect of “time from onset to seeking medical care” on “severe influenza.”

Independent variable	Mediating variable (Time from onset to antiviral treatment)					Dependent variable (Severe Influenza)				
	*B*	*t*	*p*	Adj. *R*^2^	*F*	*B*	*t*	*p*	Adj. *R*^2^	*F*
Time between onset and visit	0.061	2.149	0.032	0.103	4.619					
Time between onset and visit						0.186	5.658	<0.001	0.251	32.008
Time between onset and visit						0.126	4.101	<0.001	0.332	24.591
Time from onset to antiviral treatment						0.178	5.447	<0.001		

### 3.3 Laboratory findings of the two groups

The NLR and CRP values were greater in the severe group than in the nonsevere group, whereas the ALB value was lower in the severe group than in the nonsevere group. The differences were statistically significant (*p* < 0.001). However, there were no statistically significant differences in the other laboratory test indicators between the two groups (*p* > 0.05), as shown in Table 4.

**Table 4:** Comparison of laboratory indices between severe and nonsevere cases of influenza in Fuzhou [M (P_25_, P_75_)].

Typical symptoms	Severe group (*n* = 386)	Non severe group (*n* = 1,544)	*Z*	*p*
WBC(×10^9^/L)	5.9 (4.1,8.2)	5.5 (4.3,7.8)	1.883	0.056
Lymphocyte count (×10^9^/L)	2.7 (1.8,4.0)	2.8 (2.0,4.2)	0.654	0.531
Neutrophil count (×10^9^/L)	2.2 (1.2,3.1)	2.0 (1.3,2.8)	0.864	0.378
Monocyte count (×10^9^/L)	0.6 (0.4,0.8)	0.5 (0.3,0.7)	1.672	0.061
NLR	1.7 (0.7,2.6)	0.9 (0.5,1.4)	7.652	<0.001
CRP (mg/L)	6.9 (4.8,26.3)	5.2 (3.2,8.9)	4.583	<0.001
PCT (ug/L)	0.5 (0.2,0.7)	0.3 (0.1,0.5)	1.583	0.068
ALT (U/L)	13.5 (10.1,18.2)	12.8 (10.2,16.2)	1.073	0.127
AST (U/L)	40.1 (31.3,51.7)	39.1 (33.9,47.3)	1.268	0.103
CK-MB (U/L)	24.0 (13.8,34.2)	23.2 (19.8,26.3)	1.572	0.075
Albumin (g/L)	37.2 (35.1,39.3)	43.2 (41.2,45.3)	6.793	<0.001
Globulin (g/L)	26.9 (23.7,29.6)	25.7 (24.3,26.9)	0.788	0.476

**Figure 1:**
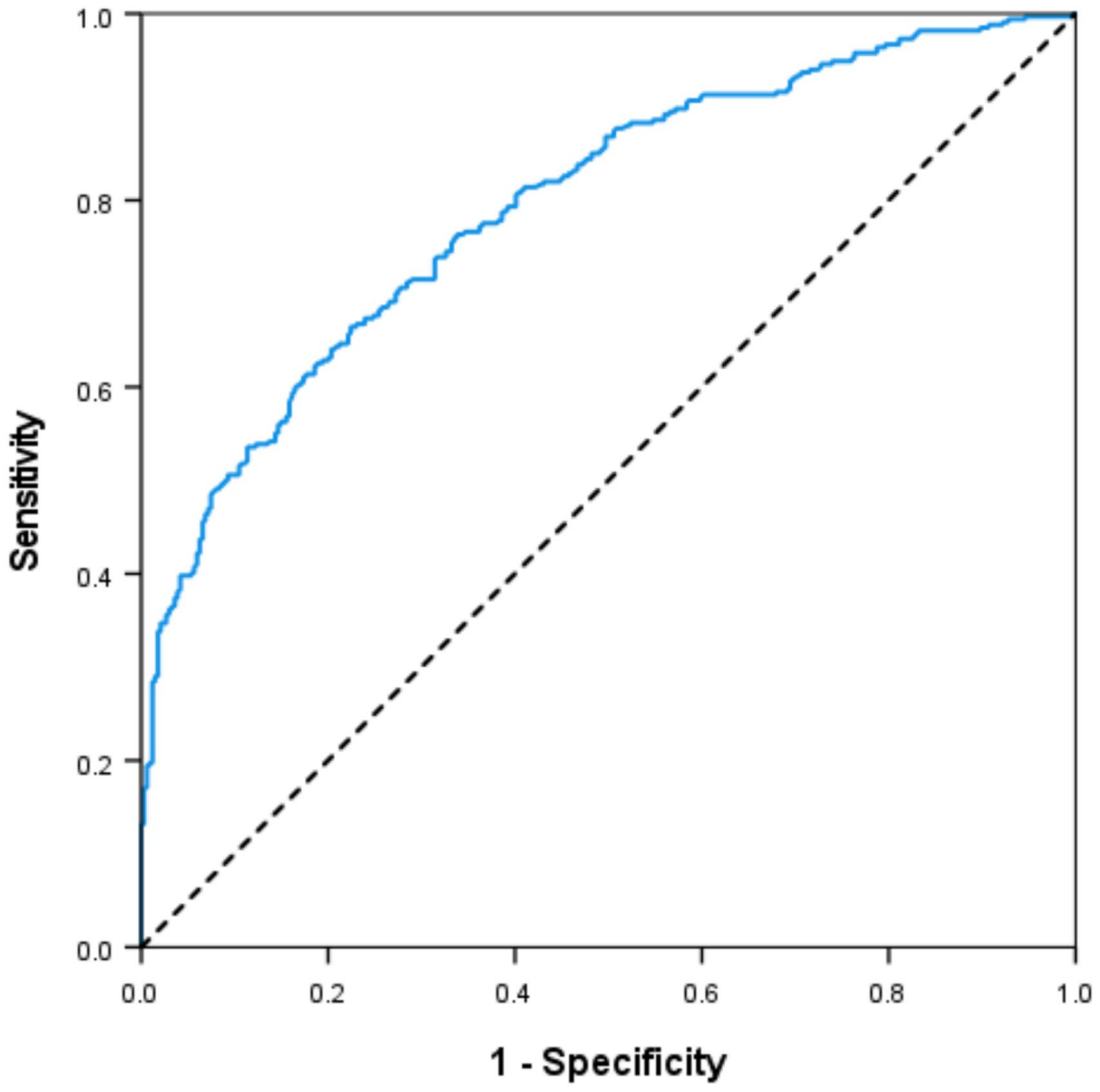
ROC curve analysis of neutrophil-to-lymphocyte ratio (NLR) for predicting severe influenza.

ROC curve analysis was performed on the NLR. The results revealed that the AUC was 0.766 (95% CI: 0.678–0.852). The Youden index was highest when the NLR was 1.52, with a sensitivity of 78.3% and specificity of 82.6% for predicting severe disease. ROC analysis (Figure 1) demonstrated that NLR predicted severe influenza with an AUC of 0.766 (95% CI: 0.678–0.852). At the optimal cutoff of 1.52 (Youden index), sensitivity was 78.3% and specificity was 82.6%.

### 3.4 Medical history of the two groups

The proportions of children with a history of chronic respiratory diseases and metabolic and endocrine system diseases were 26.17 and 16.32%, respectively, in the severe group, which were greater than the 8.35 and 9.46%, respectively, in the nonsevere group. The differences were statistically significant (*p* < 0.001). However, there were no statistically significant differences between the two groups in terms of history of cancer/tumor or surgery (*p* > 0.05), as shown in Table 5.

**Table 5:** Comparison of past medical history between severe and nonsevere cases of influenza in Fuzhou [*n* (%)].

Variable	Contents	Severe group (*n* = 386)	Non severe group (*n* = 1,544)	*χ* ^2^	*p*
History of chronic respiratory diseases	Yes	101 (26.17%)	129 (8.35%)	93.322	<0.001
	No	285 (73.83%)	1,415 (91.65%)		
History of metabolic and endocrine system diseases	Yes	63 (16.32%)	146 (9.46%)	15.072	<0.001
	No	323 (83.68%)	1,398 (90.54%)		
Cancer/tumor history	Yes	6 (1.55%)	21 (1.36%)	0.085	0.771
	No	380 (98.45%)	1,523 (98.64%)		
Surgical history	Yes	8 (2.07%)	21 (1.36%)	1.059	0.303
	No	378 (97.93%)	1,523 (98.64%)		

### 3.5 Pathogen detection of the two groups

**Figure 2:**
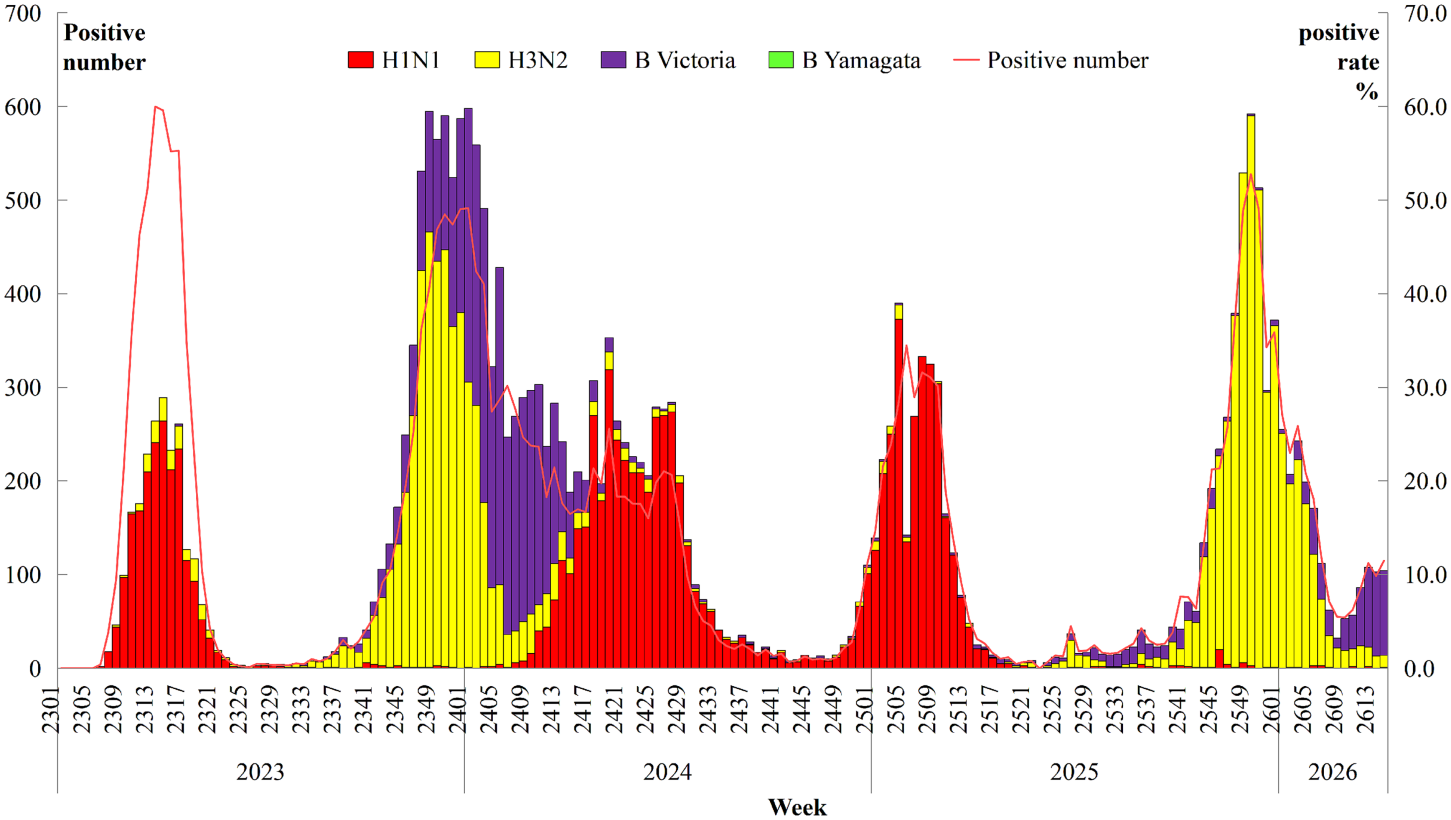
Temporal distribution of weekly influenza specimen nucleic acid testing in Fuzhou, China, 2022–2026.

With respect to virus types, the proportion of positive cases for influenza A (H1N1) was 58.55% in the severe group, which was greater than the 38.02% reported in the nonsevere group. In contrast, the proportions of positive cases for influenza A (H3N2) and influenza B were 22.80 and 18.65%, respectively, in the severe group, which were lower than the 35.56 and 26.42%, respectively, in the nonsevere group. The differences in influenza virus types between the two groups were statistically significant (*p* < 0.001), as shown in Table 6. Figure 2 shows the weekly temporal distribution of influenza nucleic acid detection in Fuzhou during 2022–2026. Distinct seasonal peaks occurred in winter–spring months, with H1N1 predominating in early 2023 and early 2025, H3N2 and B Victoria co-circulating during 2023–2024, and H3N2 peaking in late 2025.

**Table 6:** Comparison of viral serotypes between severe and nonsevere cases of influenza in Fuzhou [*n* (%)].

Virus type	Severe group (*n* = 386)	Non severe group (*n* = 1,544)	*χ* ^2^	*p*
A(H1N1)	226 (58.55%)	587 (38.02%)	53.632	<0.001
A(H3N2)	88 (22.80%)	549 (35.56%)		
B(Victoria lineage)	72 (18.65%)	408 (26.42%)		

### 3.6 Fixed effects analysis of influenza seasonality

Compared to summer, the winter risk OR value was 2.353 (95% CI = 1.730–3.205), indicating an increased risk of influenza infection progressing to severe illness during winter compared to summer (Table 7).

**Table 7:** Seasonal fixed effects analysis.

Season	Adjusted OR (95% CI)	*p*	Compared to summer
Summer	1.000	–	–
Spring	1.174 (0.984–1.383)	0.062	Increase by 15%
Autumn	1.094 (0.920–1.295)	0.285	Increase by 9%
Winter	2.353 (1.730–3.205)	<0.001	Increase by 58%

Analysis of the Interaction Effects Between H1N1 Influenza A Virus Infection and Vaccination.

Compared with non-H1N1 infection and vaccination, the OR value for H1N1 infection without vaccination was 3.775 (95% CI = 2.659–5.361). The interaction OR between the two factors was 2.437 (likelihood ratio test, *p* < 0.05), indicating an interaction effect between H1N1 infection and vaccination status. Individuals infected with H1N1 who were unvaccinated exhibited a significantly increased risk of developing severe disease (Table 8).

**Table 8:** Analysis of the interaction effects between H1N1 influenza A virus infection and vaccination.

Factor		Severe group (*n* = 386)	Non severe group (*n* = 1,544)	OR(95%CI)	Interaction	*p*
A(H1N1)	Unvaccinated					
−	−	48	341	1.000		
+	−	40	237	1.199 (0.764–1.883)		
−	+	112	616	1.292 (0.898–1.857)		
+	+	186	350	3.775 (2.659–5.361)	2.437	<0.05

### 3.7 Multivariate logistic regression analysis of severe influenza cases

The significant variables identified by the univariate analysis were included in the multivariate logistic regression analysis (Table 9). The dependent variable was whether the disease was severe (yes = 1, no = 0). The independent variables were sex (male = 1, female = 2), population characteristics (noninstitutionalized children = 1, childcare institution children = 2), obesity (yes = 1, no = 0), place of residence (rural = 1, urban = 2), vaccination (yes = 1, no = 0), interval between the onset of illness and the first medical visit (≤2 days = 1, >2 days = 2), interval between the onset of illness and the initiation of antiviral treatment (≤2 days = 1, >2 days = 2), fever duration (≤3 days = 1, >3 days = 2), tachypnea/breathing difficulty (yes = 1, no = 0), altered mental status/seizures (yes = 1, no = 0), NLR value, CRP value, albumin value, history of chronic respiratory diseases (yes = 1, no = 0), history of metabolic and endocrine system diseases (yes = 1, no = 0), and virus type (influenza B = 1, influenza A (H1N1) = 2, influenza A (H3N2) = 3). The results of the multivariate logistic regression analysis revealed that being male, being a noninstitutionalized child, being obese, living in rural areas, lacking vaccination, having an interval of more than 2 days between the onset of illness and the first medical visit, having an interval of more than 2 days between the onset of illness and the initiation of antiviral treatment, having a fever lasting more than 3 days, having tachypnea/breathing difficulty, having altered mental status/seizures, having a high NLR, having high CRP levels, having a low albumin level, having a history of chronic respiratory diseases, having a history of metabolic and endocrine system diseases, and having an infection with influenza A (H1N1) are risk factors for severe influenza in children and are likely to lead to the occurrence of severe influenza, as shown in Table 10.

**Table 9:** Collinearity assessment of risk factors.

Factor	Tolerance	Variance expansion factor
Gender	0.456	2.193
Population characteristics	0.648	1.544
Obesity or not	0.818	1.222
Current address attribution	0.766	1.306
Vaccination	0.543	1.841
Time between onset and visit	0.848	1.179
Time from onset to antiviral treatment	0.951	1.052
High fever	0.733	1.365
Shortness of breath/dyspnea	0.730	1.370
Change of mind/convulsion	0.914	1.094
High NLR	0.516	1.978
High CRP	0.525	1.904
Low albumin	0.648	1.544
History of chronic respiratory diseases	0.957	1.045
History of metabolic and endocrine system diseases	0.487	2.054
Virus type	0.900	1.111

**Table 10:** Multivariate conditional logistic regression analysis of risk factors for severe cases of influenza in Fuzhou.

Influence factor	Contents	*B*	S.E.	Wald *χ*^2^	OR	95%CI	*p*
Gender	Female				1.000		
	Male	0.413	0.178	5.389	1.511	1.066–2.141	0.020
Population characteristics	Nursery children				1.000		
	Children not enrolled in child-care institutions (home-care)	0.611	0.178	11.834	1.843	1.301–2.611	0.001
Obesity or not	No				1.000		
	Yes	0.680	0.242	7.917	1.974	1.229–3.169	0.005
Current address attribution	Urban area				1.000		
	Rural area	1.009	0.180	31.309	2.743	1.926–3.905	<0.001
Vaccinate	Yes				1.000		
	No	1.145	0.231	24.453	3.141	1.996–4.945	<0.001
Time between onset and visit	≤2d				1.000		
	>2d	0.521	0.203	6.580	1.683	1.131–2.506	0.010
Time from onset to antiviral treatment	≤2d				1.000		
	>2d	0.678	0.181	14.028	1.969	1.381–2.807	<0.001
High fever duration	≤3d				1.000		
	>3d	0.528	0.211	6.242	1.695	1.120–2.564	0.012
Shortness of breath/dyspnea		0.749	0.238	9.921	2.115	1.327–3.370	0.002
Change of mind/convulsion		1.229	0.213	33.443	3.419	2.254–5.185	<0.001
High NLR		0.950	0.184	26.567	2.585	1.801–3.709	<0.001
High CRP (mg/l)		0.752	0.172	19.054	2.121	1.513–2.973	<0.001
Low albumin (g/l)		0.695	0.179	14.995	2.004	1.410–2.849	<0.001
History of chronic respiratory diseases	No				1.000		
	Yes	1.291	0.191	45.654	3.637	2.501–5.290	<0.001
History of metabolic and endocrine system diseases	No				1.000		
	Yes	0.950	0.184	26.567	2.585	1.801–3.709	<0.001
Virus type	B (Victoria lineage)				1.000		
	A (H3N2)	0.159	0.209	0.578	1.172	0.779–1.764	0.447
	A (H1N1)	1.088	0.198	30.148	2.968	2.013–4.376	<0.001

### 3.8 Recovery times of the two groups

**Figure 3:**
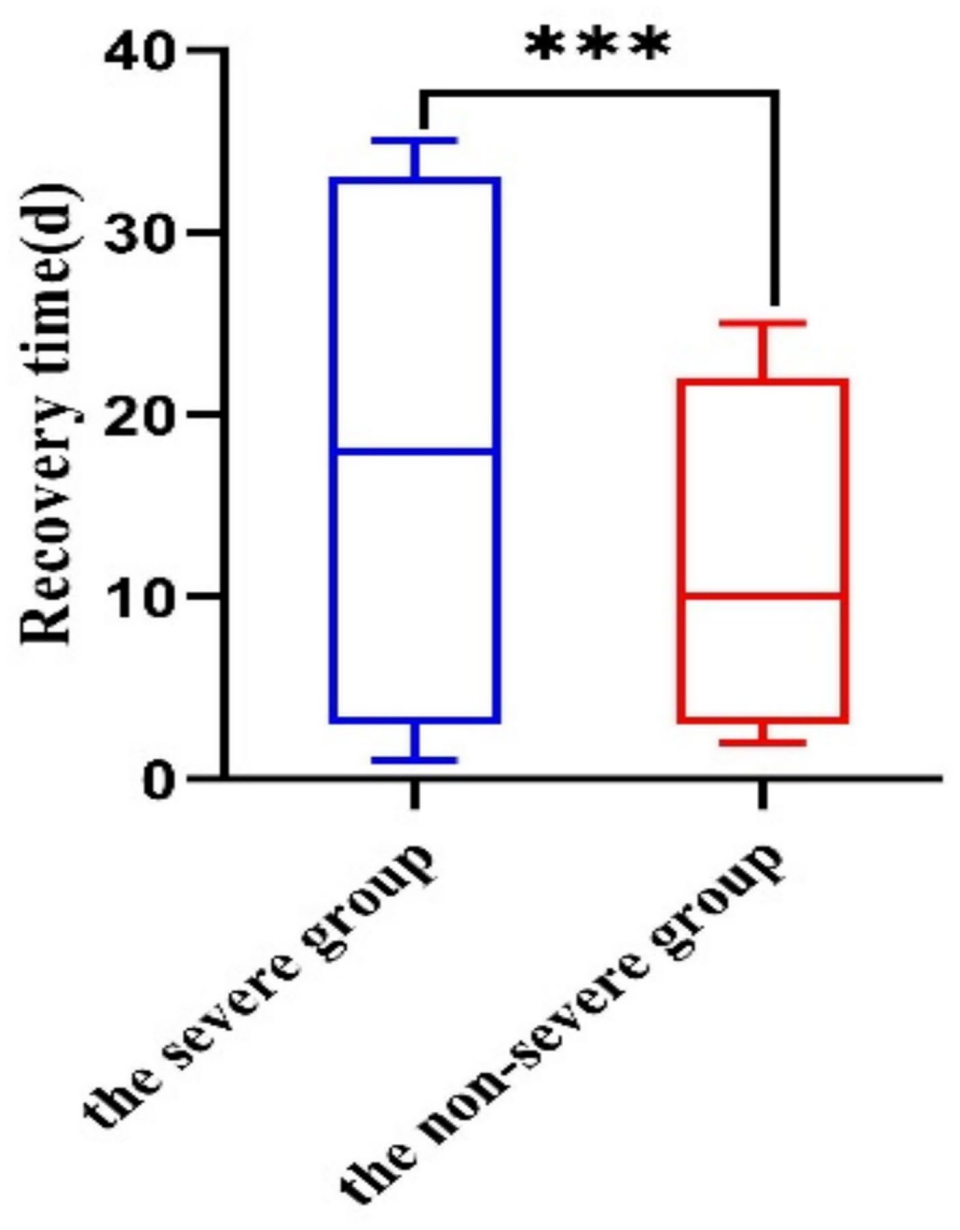
Comparison of recovery time between severe and non-severe influenza groups.

**Figure 4:**
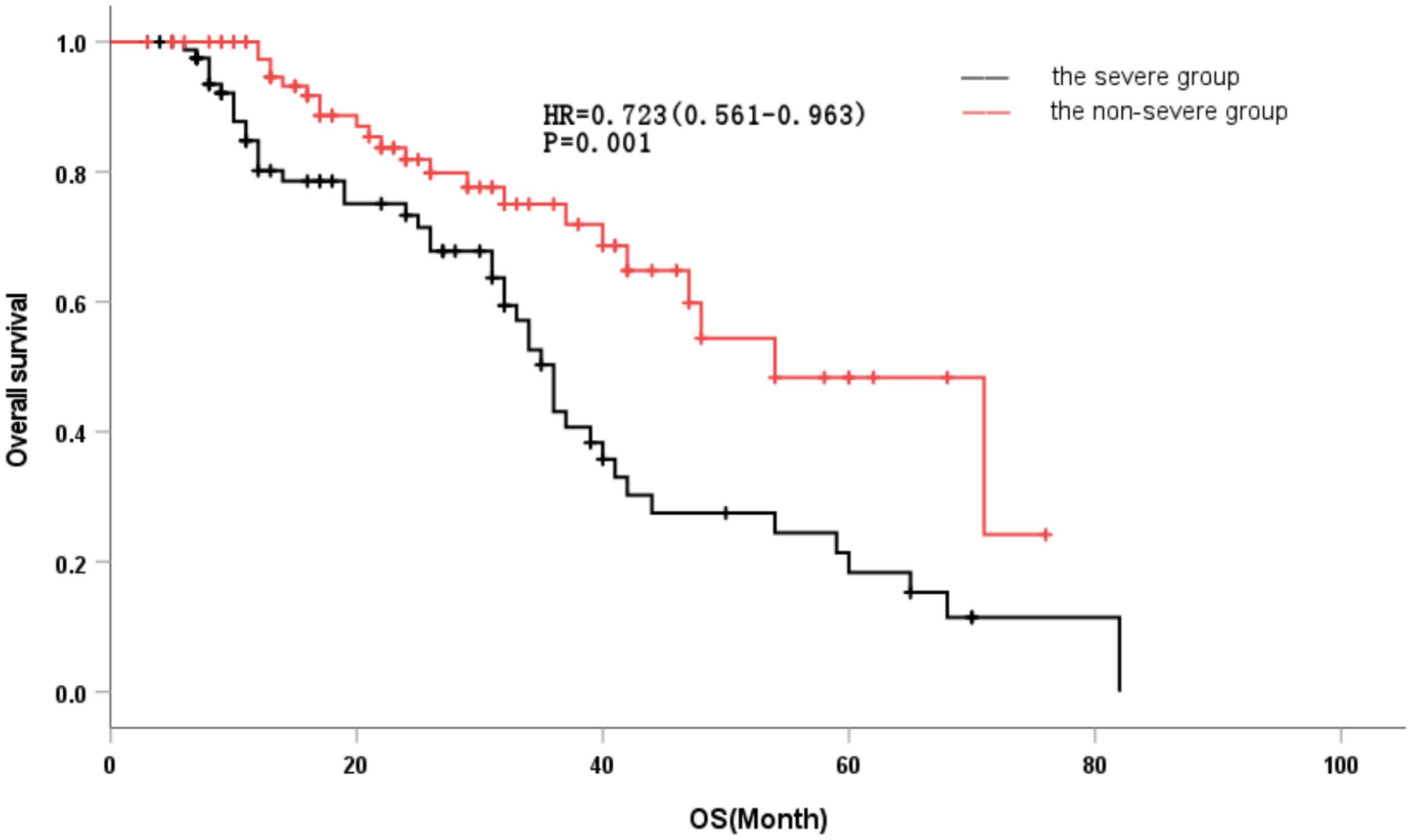
Kaplan–Meier survival curves comparing severe and non-severe influenza groups (log-rank *p* = 0.001).

The median recovery time was 19 (4, 32) days in the severe group and 10 (4, 24) days in the nonsevere group, indicating a significant difference (*Z* = 8.687, *p* < 0.001). As shown in Figure 3, the severe group had significantly prolonged recovery time compared to the non-severe group (median 19 vs. 10 days, *p* < 0.001). Kaplan–Meier analysis (Figure 4) showed significantly worse overall survival in the severe group compared to the non-severe group (HR = 0.723, 95% CI: 0.561–0.963; *p* = 0.001).

## 4 Discussion

Influenza is a key infectious disease targeted for prevention and control in Fuzhou city, with children being particularly susceptible. The progression to severe disease can significantly endanger the lives of affected children and is one of the leading causes of death from respiratory diseases in children (8). This study collected data on 386 severe and 1,544 nonsevere influenza cases in children under the age of 5, who were residents of Fuzhou city and experienced illness between January 2015 and January 2025, to analyze the risk factors for severe influenza. The findings revealed that in terms of demographic characteristics, the proportion of males was greater in the severe group than in the nonsevere group, indicating that male sex is a risk factor for severe influenza in children. Robbins et al. (9) reported that boys have significantly higher rates of severe illness and hospitalization than girls do. They suggested that when infected with the virus, girls tend to mount a faster and stronger immune response, producing more interferons and antibodies, which helps prevent the development of severe pneumonia. In contrast, boys’ innate immunological disadvantage results in a relatively weaker immune response to influenza virus infection, making them more prone to severe disease (10). The proportion of children living in noninstitutional settings was also greater in the severe group than in the nonsevere group. Children in noninstitutional settings are primarily at risk due to their young age, immature immune systems, and lower levels of IFN-*γ* and IL-2, which result in weaker T-cell activation and consequently milder immune responses (11). Additionally, these children often lack good hygiene practices, such as frequent object touching and finger-sucking, which increases the risk of viral infections and severe disease progression (12). Furthermore, the absence of regular health check-ups (e.g., morning and afternoon examinations) in these settings further elevates the likelihood of severe outcomes (13). Additionally, the proportion of obese children was greater in the severe group than in the nonsevere group. Kammerl et al. (14) demonstrated that obesity can alter cellular immunity and reduce the body’s immune response to the influenza virus. Brits et al. (15) reported that obesity can prolong the shedding time of the influenza A (H1N1) virus, increasing the risk of complications and death. Moreover, the proportion of children residing in rural areas was greater in the severe group than in the nonsevere group. Liao et al. (16) reported that compared with urban areas, rural regions have poorer living conditions and sanitation. Limited medical resources and less convenient access to healthcare for children in rural areas, coupled with the lower medical standards and diagnostic capabilities of rural healthcare facilities, contribute to a greater risk of severe influenza in these areas. The vaccination rate was lower in the severe group than in the nonsevere group. This is consistent with previous research findings, indicating that vaccinated children typically exhibit milder symptoms when infected with the virus, while unvaccinated children are more prone to developing severe cases (17). The overall influenza vaccination rate among children in Fuzhou city is relatively low. Therefore, promoting vaccination among eligible children can effectively reduce the incidence of severe disease.

Clinically, a delay of more than 2 days between the onset of illness and medical consultation is a risk factor for severe influenza. This is because early detection and timely treatment can effectively curb viral replication and prevent the progression to severe disease (18). Therefore, it is essential to closely monitor children’s health status. Prompt medical attention following the onset of illness can significantly reduce the risk of severe disease. Similarly, a delay of more than 2 days between the onset of illness and the initiation of antiviral treatment is also a risk factor for severe influenza. Yang et al. (19) demonstrated that antiviral treatment within 2 days of the onset of influenza-like symptoms can effectively alleviate symptoms and reduce the severity rate, highlighting the importance of early antiviral therapy. A persistent high fever lasting more than 3 days is another risk factor for severe influenza, as concluded by Zhou (20). Children with influenza who have a fever lasting more than 3 days are more likely to develop respiratory complications. The reason is that children with more severe infections often experience fever, which tends to worsen as the disease progresses. A persistent high fever can easily lead to pneumonia and subsequently severe disease. Therefore, children with influenza and a high fever lasting more than 3 days should be closely monitored and receive timely interventions such as antipyretic treatment. This study also identified tachypnea/dyspnea as a risk factor for severe disease. The presence of these symptoms indicates significant inflammatory changes in the lungs. As lung damage progresses, respiratory distress syndrome may develop, leading to severe disease (21). Additionally, altered mental status/seizures were found to be risk factors for severe disease, consistent with the findings of Edsen (22). The proportion of children with altered mental status/seizures was higher in the severe group than in the nonsevere group. This is because the influenza virus can invade the blood–brain barrier, leading to neurological complications. The presence of altered mental status/seizures in influenza patients often indicates a more severe stage of the disease (23). Children with influenza and neurological complications also consume more medical resources and have higher mortality rates (24). Therefore, this study emphasizes the importance of timely medical consultation following the onset of influenza-like symptoms, early antiviral treatment, and special attention to children with influenza who have a high fever lasting more than 3 days, tachypnea/dyspnea, or altered mental status/seizures to prevent the progression to severe disease.

In terms of laboratory tests, this study found that the neutrophil-to-lymphocyte ratio (NLR) was significantly higher in the severe group than in the nonsevere group, indicating that a high NLR is a risk factor for severe disease. ROC analysis revealed that an NLR value of 1.52 has a sensitivity of 78.3% and a specificity of 82.6%, making it a valuable predictor of severe influenza. This aligns with previous research findings, indicating that NLR serves as an independent predictor for severe influenza and holds significant value in identifying high-risk populations for influenza (25). The study also found that C-reactive protein (CRP) levels were higher in the severe group than in the nonsevere group, suggesting that elevated CRP is a risk factor for severe disease. A previous study also indicated that elevated CRP levels are associated with the severity of influenza (26). Therefore, monitoring changes in CRP levels can help assess the condition of affected children. Additionally, albumin levels were significantly lower in severe cases, indicating that low albumin levels are associated with an increased risk of severe disease. Xie et al. (27) discovered that albumin can serve as a prognostic indicator, with low albumin levels being a risk factor for severe influenza in children, while high albumin levels are protective. They recommended early albumin infusion for children with severe influenza.

Regarding past medical history, this study found that the proportion of children with a history of chronic respiratory diseases was higher in the severe group than in the nonsevere group, consistent with the findings of Sopori (28). He suggested that individuals with a history of chronic respiratory diseases have a lower proportion of T cells and compromised immune function. Moreover, these individuals often have multiple lobular and segmental inflammatory lesions in the lungs. When infected with the influenza virus, these preexisting conditions can exacerbate lung damage and lead to severe disease. The study also found that the proportion of children with a history of metabolic and endocrine system diseases was higher in the severe group than in the nonsevere group, in line with the findings of Luo (29). Children with a history of metabolic and endocrine system diseases have a lower proportion of T cells and weaker immune responses compared to healthy children, making them more susceptible to severe disease following viral infection.

In terms of viral subtypes, this study identified that infection with influenza A (H1N1) is a risk factor for severe disease in children, consistent with the findings of Mei (30). Compared to influenza B, influenza A (H1N1) is more likely to impair the body’s ability to clear the virus. The virus continues to replicate in the body, persistently infecting the alveoli and bronchi, thereby worsening lung damage. Fu et al. (31) discovered that the influenza A (H1N1) virus can promote the release of a large number of inflammatory factors, such as PAI-1, uPA, and tPA, and induce cell apoptosis. This leads to more severe damage to human bronchial epithelial cells and lung tissue. Therefore, infections caused by the influenza A (H1N1) virus tend to result in more severe illness and should be taken seriously. The study also found that the recovery time for severe patients was 19 (4, 32) days, which is longer than that for nonsevere patients. Therefore, medical institutions at all levels should actively provide case treatment and timely referrals to prevent delays in treatment that could lead to severe disease.

To effectively prevent and control severe influenza in children in our city, the following recommendations are proposed: First, focus on epidemic prevention and control among key populations. To safeguard the health of vulnerable groups, it is essential to prioritize the prevention and control of influenza among children. Second, strengthen prevention and control measures in childcare institutions by strictly implementing morning and afternoon health checks and recording absences due to illness. Childcare institutions should promptly register cases and enforce epidemic reporting protocols. Third, enhance vaccination promotion and continuously advance influenza vaccination among eligible children, starting at 6 months of age. Additionally, efforts should be made to increase the vaccination rate among family members. Influenza vaccination can effectively prevent influenza-related diseases and significantly reduce the risk of outpatient visits and hospitalizations among children and adolescents (32, 33). Fourth, promote the allocation of medical resources to rural areas and improve diagnostic and treatment capabilities. Currently, rural areas are a crucial front for the prevention and control of severe hand, foot, and mouth disease. However, there is still a significant shortage of technical personnel and medical supplies in these areas. Therefore, technical personnel from county-level cities should be redirected to weaker township health centers to supplement technical strength and enhance treatment capabilities. At the same time, the allocation of emergency equipment and medications should also be tilted toward township health centers. Fifth, strengthen the training of medical staff to improve the treatment level of severe cases. Timely referrals to higher-level hospitals should be made when necessary to ensure early detection, intervention, referral, and treatment (34), thereby reducing the risk of severe disease.

This study also has several limitations. First, the case–control study design inevitably introduces recall bias. There may be inadequate consideration of confounding factors during the matching process, and selection bias may occur when choosing controls. Second, although case information was collected through two disease control information systems, these systems may still have underreporting or misreporting issues, which could affect the study conclusions. Third, this study examined potential risk factors from multiple dimensions, including clinical symptoms and laboratory tests. Given the multitude of factors involved and the difficulty in controlling their interactions, the conclusions may exhibit some bias. Furthermore, as the provincial capital, Fuzhou’s resident characteristics and healthcare conditions may differ from other regions, limiting the generalizability of the findings and the applicability of the conclusions.

## 5 Conclusion

This study identified multiple risk factors associated with severe influenza in children. These factors include: male gender, children not enrolled in child-care institutions (home care) living environment, obesity, rural residence, lack of vaccination, delayed medical care and antiviral treatment, persistent high fever, shortness of breath/ dyspnea, altered mental status/seizures, elevated neutrophil-to-lymphocyte ratio (NLR) and C-reactive protein (CRP), low albumin levels, history of chronic respiratory disease, history of metabolic and endocrine disorders, and infection with influenza A (H1N1) virus. These findings underscore the complexity of severe influenza in children and its multifactorial pathogenesis. Future efforts should focus on targeted interventions: controlling the aforementioned risk factors, increasing vaccination coverage, refining early diagnosis and treatment protocols, and strengthening healthcare infrastructure (particularly in rural areas). These measures hold promise for reducing influenza incidence and severity in children, ultimately improving their health outcomes.

## Data Availability

The original contributions presented in the study are included in the article/supplementary material, further inquiries can be directed to the corresponding authors.
